# Hyperspectral Imaging for Presymptomatic Detection of Tobacco Disease with Successive Projections Algorithm and Machine-learning Classifiers

**DOI:** 10.1038/s41598-017-04501-2

**Published:** 2017-06-23

**Authors:** Hongyan Zhu, Bingquan Chu, Chu Zhang, Fei Liu, Linjun Jiang, Yong He

**Affiliations:** College of Biosystems Engineering and Food Science, Zhejiang University, Hangzhou, 310058 China

## Abstract

We investigated the feasibility and potentiality of presymptomatic detection of tobacco disease using hyperspectral imaging, combined with the variable selection method and machine-learning classifiers. Images from healthy and TMV-infected leaves with 2, 4, and 6 days post infection were acquired by a pushbroom hyperspectral reflectance imaging system covering the spectral range of 380–1023 nm. Successive projections algorithm was evaluated for effective wavelengths (EWs) selection. Four texture features, including contrast, correlation, entropy, and homogeneity were extracted according to grey-level co-occurrence matrix (GLCM). Additionally, different machine-learning algorithms were developed and compared to detect and classify disease stages with EWs, texture features and data fusion respectively. The performance of chemometric models with data fusion manifested better results with classification accuracies of calibration and prediction all above 80% than those only using EWs or texture features; the accuracies were up to 95% employing back propagation neural network (BPNN), extreme learning machine (ELM), and least squares support vector machine (LS-SVM) models. Hence, hyperspectral imaging has the potential as a fast and non-invasive method to identify infected leaves in a short period of time (i.e. 48 h) in comparison to the reference images (5 days for visible symptoms of infection, 11 days for typical symptoms).

## Introduction

As a model plant, tobacco (*Nicotiana tabacum* L.) has high economic value and comprehensive usage with short growth period and specific morphology characteristics. However, the quality and yield of tobacco can be easily affected by various diseases during the growing season. Tobacco mosaic virus (TMV) is a devastating worldwide disease, which can cause major yield reduction and economic losses in agricultural industry. It is crucial to accurately identify the TMV-infected plants at an early stage to mitigate the worldwide losses for sustainable agriculture which aims to target chemicals where and when needed at an appropriate dose^1^. Conventionally, detecting diseases on crop leaves is based on direct and indirect methods^2^. Direct methods are mainly visual assessments, serological and molecular methods^3^, including flow cytometry, enzyme-linked immune sorbent assay (ELISA), immunofluorescence (IF), polymerase chain reaction (PCR), and fluorescence *in situ* hybridization (FISH). Indirect methods are based on biomarker-based detection technology (i.e. gaseous metabolite profiling and plant metabolite profiling) and plant properties/stress. Unfortunately, these techniques for the identification of plant diseases are time-consuming, inefficient and destructive, which also require detailed sampling and processing procedures^2, 4^. Additionally, highly trained and qualified technicians are necessary for sophisticated detection techniques. Therefore, an advanced and time-efficient method is necessary for early information on disease detection in a large scale of fields, which can facilitate the control of diseases through proper management strategies and improve productivity.

Originally developed for remote sensing applications, hyperspectral imaging (HSI), also known as spectroscopic or chemical imaging, has witnessed a tremendous growth of its applications in diverse fields^5–7^. Compared with conventional RGB imaging, near infrared (NIR) spectroscopy and multispectral imaging, HSI integrates conventional imaging and spectroscopy to obtain both spatial and spectral information simultaneously from a sample at spatial resolutions varying from the level of single cells up to macroscopic objects^6, 8, 9^. Known as hypercubes, hyperspectral images are three-dimensional blocks of data, comprising two spatial and one wavelength dimensions. The spectral signature of leaves results from multiple interactions between incoming irradiation and biophysical (e.g. leaf surface, tissue structure) and biochemical characteristics (e.g. the content of pigments and water)^10^. In particular, the reflectance at visible wavelengths provides information on leaf pigments, while the reflectance at infrared wavelengths represents the physiological condition of the plant^2, 11^.

A grow body of research has investigated the chemical components such as nitrogen (N)^7^, phosphorus (P), potassium (K)^8^, and soluble protein^12^, in certain organ tissue (e.g. leaves) of crops. Meanwhile, much attention has been attracted towards using hyperspectral imaging technique for the disease detection of precision agriculture-based applications^13, 14^. Diseases can result in changes of transpiration rate, morphology, leaf color, and crop density, which in turn affect the optical properties of the plants. Del Fiore *et al*.^15^ demonstrated that the hyperspectral imaging was able to rapidly discriminate commercial maize kernels infected with toxigenic fungi from healthy leaves using only spectral reflectance features. Mahlein *et al*.^10^ differentiated three sugar beet diseases (cercospora leaf spot, powdery mildew and leaf rust) with hyperspectral imaging technique and spectral angle mapper. Xie *et al*.^4^ investigated the possibility of utilizing hyperspectral imaging for detecting different diseases (early blight and late blight) on tomato leaves. Extreme learning machine model was developed for the classification of healthy, early blight and late blight of detached leaves, which yielded about 97.1–100% classification accuracy when classifying diseased plants based on spectral information. The texture analysis achieved the accuracy of about 70%. Nevertheless, the aforementioned researches always used a common machine-learning model^13^ based on spectral signature ignoring the importance of data fusion^4^. Few literatures are comprehensively conducted on differentiating the infected and non-infected leaves in an early stage and classifying different degrees of disease using HSI with spectral analysis, texture analysis and data fusion.

Consequently, the main purpose of this work was to establish a methodology for presymptomatic detection of tobacco disease based upon HSI. This outmost goal was achieved by meeting the following specific objectives: (i) determining the corresponding effective wavelengths (EWs) which give the highest correlation between the spectral data and different disease stages; (ii) extracting texture features based on grey-level co-occurrence matrix (GLCM) at the selected EWs; (iii) developing and comparing robust and accurate machine-learning models with spectral data, texture features and data fusion respectively, to quantitatively identify the tobacco disease; (iv) discriminating TMV-infected from non-diseased tobacco leaves and classifying three levels of disease degree during the infected period even before specific symptoms became visible.

## Results

### Characteristics of spectral profiles

**Figure 1:**
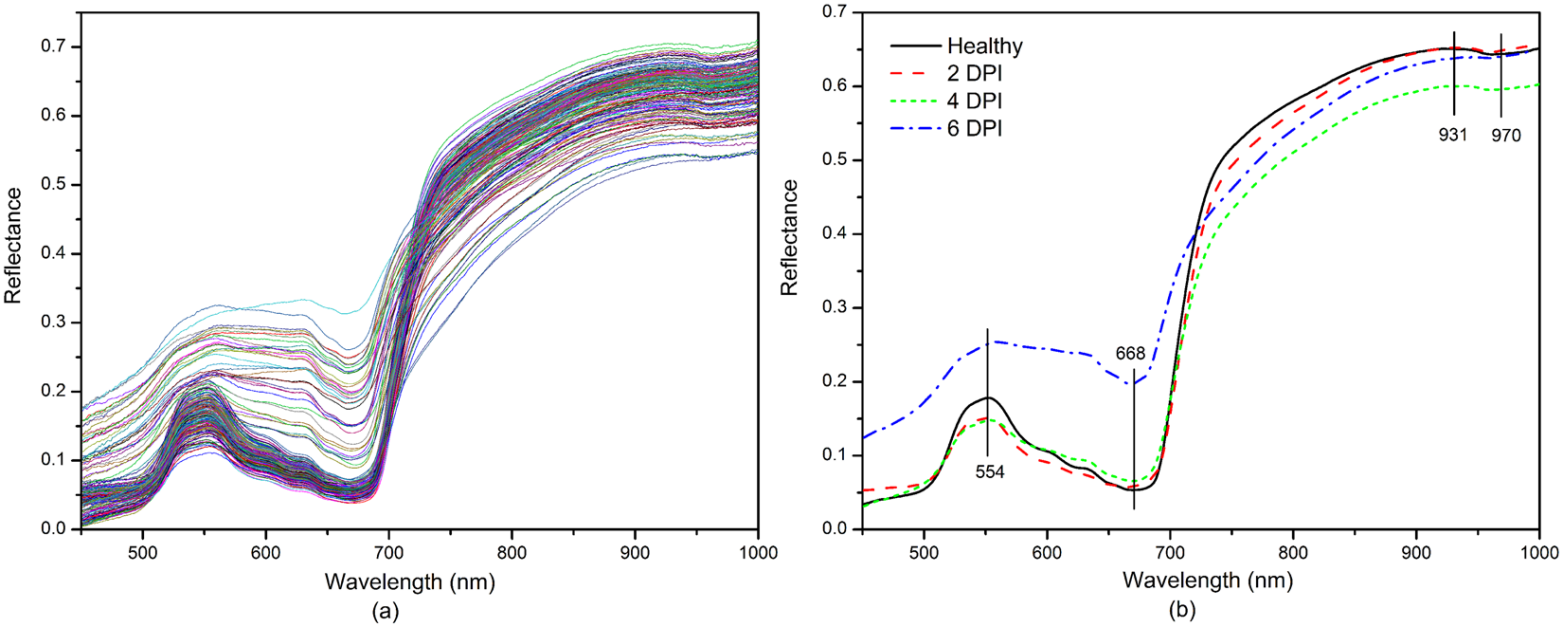
(**a**) Raw spectra (450–1000 nm) of tobacco leaves samples; (**b**) The average reflectance spectra of ‘Healthy’, ‘2 DPI’, ‘4 DPI’, and ‘6 DPI’ tobacco leaves at 450–1000 nm.

There are 512 bands (variables) covering the Vis/NIR spectral range (380–1023 nm). Nonetheless, only spectra in 450–1000 nm were used for analysis since the beginning and ending of the wavelengths revealed obviously noisy signals. Figure 1a displays the mean spectra of ROIs extracted from 180 hyperspectral images. The pattern of the reflectance curves was similar to that for other green plant leaves such as oilseed rape^12^, maize^16^ and tomato^4^ although the position and magnitude of the valleys were specific. The average reflectance spectra of healthy (no treatment), 2 days post infection (DPI), 4 DPI, and 6 DPI tobacco leaves at 450–1000 nm spectral range with a total of 434 bands are illustrated by Fig. 1b. The general trends of the four spectral curves were quite similar. There was a peak at around 554 nm and a valley at around 668 nm. The reflectance increased sharply with the wavelengths from about 670 to 750 nm, and was kept at a high level between 750 nm and 900 nm. According to Weber *et al*.^16^ and Zhang *et al*.^8^, the reflectance is associated with the photosynthetic capacity (495–680 nm), red inflection point (680–780 nm) and plant water status (970 nm).

As detailed in Fig. 1b, there were no visibly noticeable differences between the healthy and the inoculated leaves with 2 DPI, which revealed the difficulty of detecting and classifying plant-virus interactions at early stages. Reflectance of healthy samples was higher than that of infected ones (2 DPI, 4 DPI, and 6 DPI) in the spectral window of 750–1000 nm (Fig. 1b), which was probably due to the collapse of leaf cell structure with the disease spread. However, it was challenging to visually differentiate the TMV-infected leaves from healthy samples based on the spectra covering the entire wavelength region of 450–1000 nm. The visual symptoms of infection occurred after 5 days and the systemic symptoms appeared on the 11th day. Thereupon, large increases in the reflectance of diseased samples (6 DPI) over the spectral region of 450–700 nm were observed, which could be attributed to the combined effect of discoloration and tissue damage.

### Selection of effective wavelengths

Hyperspectral images, as high-dimensional data, have a high degree of inter-band correlation, resulting in information redundancy which can cause convergence instability in the multivariate prediction models. Hence, the application of fewer wavebands is preferable for more stable models and easier implementation in the subsequent multispectral imaging system^17^. The selected EWs which carried the most information for early detection and classification of TMV-infected tobacco leaves could be used as the direct input of the machine-learning models.

**Figure 2:**
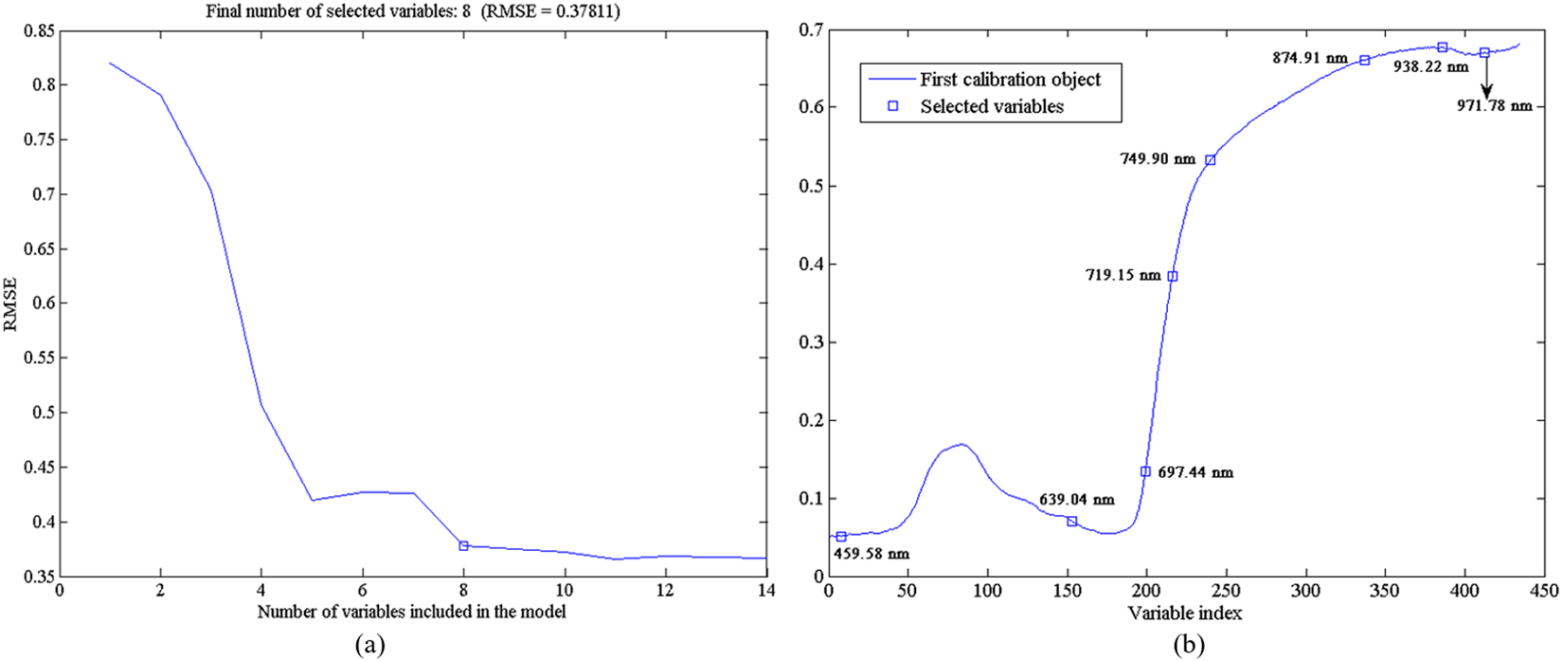
Selection of effective wavelengths by SPA: (**a**) final number of selected variables according to the minimum root mean square error of validation (RMSEV) in the validation set of multiple linear regression (MLR) calibration; (**b**) effective wavelengths shown in circle markers.

Herein, successive projections algorithm (SPA), which was conducted by a series of MATLAB programs, was proposed to select EWs from 434 variables. As illustrated by Fig. 2, eight EWs which were relevant to the detection of tobacco leave disease at an early stage (697.44, 639.04, 938.22, 719.15, 749.90, 874.91, 459.58, and 971.78 nm) were determined based on the minimum root mean square error of validation (RMSEV) in the validation set of multiple linear regression (MLR) calibration. Furthermore, the selected EWs by SPA were sequenced in the order of relevance and then used to replace the full wavelengths for identification of different diseases. The wavelength number was decreased by more than 98% ($\frac{434-8}{434}=98.16 \%$) after wavelengths selection by SPA, which influentially simplified the calibration models and reduced the computation complexity. The selected EWs (459.58 nm) might be associated with the absorption of anthocyanin. In addition, chlorophyll-*b* (620 nm) and chlorophyll-*a* (675 nm) showed characteristic peaks, which could explain the selected EWs of 697.44 nm and 639.04 nm to some extent. The wavelengths of 938.22 nm and 971.78 nm were assigned to the first and second O-H stretching overtones. The above wavelength assignment demonstrated that SPA was quite useful for the selection of relevant variables.

### Machine-learning classification with EWs

As a consequence of the previous analysis, wavelength selection method prominently reduced the number of wavelengths. The selected EWs instead of full spectra were then applied to establish calibration models. To detect the stage of disease accurately and acquire more information, different machine-learning methods were applied and compared in the development of calibration models. The selected EWs by SPA were used as the direct input of the machine-learning algorithms. A broad set of classifiers were evaluated, including partial least squares-discrimination analysis (PLS-DA), random forest (RF), support vector machine (SVM), back propagation neural network (BPNN), extreme learning machine (ELM) and least squares support vector machine (LS-SVM). The overall and individual (healthy, 2 DPI, 4 DPI or 6 DPI) classification accuracies of developed models with the abovementioned EWs are presented and compared in Table 1. The newly proposed combination of models (SPA-PLS-DA, SPA-RF, SPA-SVM, SPA-LS-SVM, SPA-ELM, and SPA-BPNN) were evaluated and compared. It can be observed from Table 1 that the performances upon different models varied from one another. The overall classification accuracies of the calibration set and the prediction set for different classifiers varied between 84.17–100.00% and 75.00–98.33%, respectively.

**Table 1:** Overall and individual class classification accuracies of machine-learning classifiers with effective wavelengths. ^a^

Classifier	Parameter	Calibration set accuracy (%)					Prediction set accuracy (%)				
		Healthy	2 DPI	4 DPI	6 DPI	Overall	Healthy	2 DPI	4 DPI	6 DPI	Overall
SPA-PLS-DA	6	95.00	95.00	50.00	75.00	84.17	80.00	90.00	50.00	70.00	75.00
SPA-RF	71	100.00	100.00	100.00	100.00	100.00	80.00	90.00	90.00	90.00	85.00
SPA-SVM	(0.01, 48.50)	100.00	100.00	95.00	95.00	98.33	86.67	90.00	80.00	90.00	86.67
SPA-LS-SVM	(33.87, 2.39)	100.00	100.00	100.00	100.00	100.00	90.00	80.00	90.00	90.00	88.30
SPA-ELM	44	100.00	100.00	100.00	100.00	100.00	96.67	100.00	100.00	100.00	98.33
SPA-BPNN	5	100.00	100.00	100.00	100.00	100.00	93.33	100.00	100.00	100.00	96.67

^a^Parameter: number of LVs for partial least squares-discrimination analysis (PLS-DA), number of forest trees for random forest (RF), (C, g) for support vector machine (SVM), (γ, σ^2^) for least squares support vector machine (LS-SVM), number of nods for extreme learning machine (ELM), and number of neurons in the hidden layer for back propagation neural network (BPNN).

In brief, the PLS-DA model obtained relatively worse performance than the other models, with a slightly low accuracy (75.00%). The majority of the machine-learning classifiers gave satisfactory results with the classification accuracies of the prediction set over 85%. Moreover, the ELM and BPNN models successfully detected the healthy and diseased tobacco leaves (2 DPI, 4 DPI, and 6 DPI) with their overall classification accuracies as high as 98.33% and 96.67% respectively. It was reasonable to assume that healthy, 2 DPI, 4 DPI, and 6 DPI leaves covering a large scope were good to develop accurate and robust machine-learning models. Therefore, ELM and BPNN with the hyperspectral imaging sensor might be more applicable in detecting TMV-infected leaves under laboratory conditions.

### Classification based on image analysis using textural features

**Figure 3:**
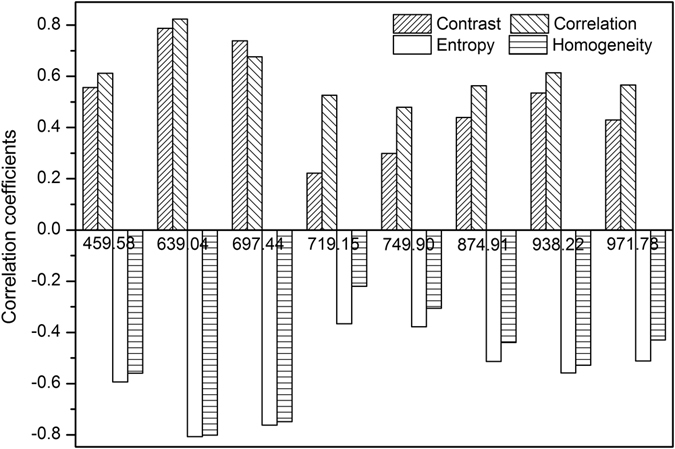
Correlation coefficients between reference values of disease degree and texture variables from effective wavelengths.

The hyperspectral cube (hyperspectral image) contains a wealth of gray-scale images at continuous wavelengths. Extracted texture features from each gray-scale image result in information redundancy which is difficult to process and not beneficial to identify the tobacco disease at the early stage. Thereupon, we extracted four kinds of textural features (contrast, correlation, entropy, and homogeneity) from the hyperspectral images only at the selected EWs according to GLCM method. Pearson correlation (*r*) was used to examine the relationship between texture variables and reference values of stages (healthy, 2 DPI, 4 DPI, and 6 DPI leaves). As can be seen from Fig. 3, significant correlations exist between the reference values and the features of contrast, correlation, entropy as well as homogeneity at selected wavelengths (P ≤ 0.01). Additionally, the correlations were mostly more than 0.5, especially at 639.04 nm and 697.44 nm, which demonstrated that the four textural features all be useful for discriminating TMV-infected from non-diseased tobacco leaves and classifying three levels of disease degree. A growing body of research has demonstrated that the classification results of texture fusion are generally higher than those obtained with the individual features^4, 18, 19^. Furthermore, texture fusion often refers to the combination of popular textural features derived from the GLCM instead of individual feature or the combination of two or three of them according to extensive literatures^19–21^.

Herein, a new dataset with 32 texture variables (4 texture features × 8 wavelengths) were obtained from the ROI for each sample, which were treated as the inputs to establish machine-learning classifiers. Different machine-learning models were built on the extracted texture features, and the results were presented in Table 2. On the whole, most classification models performed well, among which BPNN performed better than other models, with its classification accuracies of calibration and prediction reaching 95.00% and 93.33%, respectively. Although results acquired by texture analysis based on GLCM are a little lower than those obtained by spectral reflectance, the overall results indicated that it was feasible and quite promising to use texture feature for identifying the tobacco disease with regard to different developmental disease stages.

**Table 2:** Overall and individual class classification accuracies of machine-learning models with texture features. ^a^

Classifier	Parameter	Calibration set accuracy (%)					Prediction set accuracy (%)				
		Healthy	2 DPI	4 DPI	6 DPI	Overall	Healthy	2 DPI	4 DPI	6 DPI	Overall
PLS-DA	2	85.00	95.00	50.00	60.00	76.67	90.00	100.00	60.00	50.00	80.00
RF	75	100.00	100.00	100.00	100.00	100.00	93.33	50.00	80.00	100.00	85.00
SVM	(0.01, 3.03)	100.00	100.00	100.00	100.00	100.00	96.67	60.00	70.00	100.00	86.67
LS-SVM	(1.48, 22.34)	100.00	90.00	90.00	100.00	96.67	100.00	30.00	70.00	100.00	83.33
ELM	42	100.00	95.00	90.00	100.00	97.50	93.33	90.00	70.00	100.00	90.00
BPNN	10	98.33	85.00	90.00	100.00	95.00	96.67	90.00	80.00	100.00	93.33

^a^Parameters and abbreviations as in Table 1.

### Calibration models and prediction performance with data fusion

As outlined in the introduction, previous researches were mainly focused on utilizing spectra data. On the other hand, from the above analysis, the results showed that both spectral information and texture features could detect and evaluate the stage of diseased development. Data fusion is a combination of spectral data and texture variables, which has been proposed in previous literatures and mostly used in food safety and inspection^21–24^. As detailed in Table 3, the prediction performances of different machine-learning models upon data fusion were summarized.

**Table 3:** Classification results of machine-learning models with data fusion combining optimal wavelengths and texture features. ^a^

Classifier	Parameter	Calibration set accuracy (%)					Prediction set accuracy (%)				
		Healthy	2 DPI	4 DPI	6 DPI	Overall	Healthy	2 DPI	4 DPI	6 DPI	Overall
PLS-DA	2	93.33	100.00	45.00	65.00	81.67	90.00	90.00	80.00	50.00	81.67
RF	62	100.00	100.00	100.00	100.00	100.00	93.33	90.00	90.00	100.00	93.33
SVM	(1.00, 1.00)	100.00	80.00	80.00	100.00	93.33	93.33	80.00	70.00	100.00	88.33
LS-SVM	(33.52, 9.88)	100.00	100.00	100.00	100.00	100.00	100.00	90.00	90.00	100.00	96.67
ELM	60	100.00	100.00	100.00	100.00	100.00	96.67	90.00	70.00	90.00	90.00
BPNN	6	98.33	100.00	95.00	100.00	98.33	96.67	90.00	90.00	100.00	95.00

^a^Parameters and abbreviations as in Table 1.

PLS-DA model was built with the combined data as *X* and the category values as *Y* adopting the leave-one-out cross validation. The threshold value of the PLS-DA model was set equal to 0.5. The optimal number of latent variables (LVs) was determined by the minimum *Y* residual variance. The classification accuracies of the training set and the test set were 81.67% and 81.67% with 2 LVs. For BPNN model, the learning rate was 0.1 and the iteration epochs were 1000. The number of neurons of the hidden layer was set from 3 to 10. After comparing the performances of BPNN models with different number of neurons in the hidden layer, the optimal number was determined as 6 with a success rate of 95.00%. The number of nodes in the hidden layer was influential within ELM models. The optimal number of nodes in the hidden layer was based upon a stepwise search. The number of nodes was selected from 1 to 120 with a step of 1. The optimal result was obtained by 60 nodes in the hidden layer. For RF model, the number of trees in the forest was set from 50 to 200, and the number of features to be used for each node was 5. The optimal number of trees was determined by the performances of RF models. The optimal parameter of forest trees was 62 with the accuracies of the training set and the test set up to 100.00% and 93.33% separately. With regard to SVM model, the optimal penalty coefficient (C) and the kernel function parameter gamma (g) were obtained by a grid-search procedure in the range of 2^−8^–2^8^ with the kernel function of RBF. In terms of LS-SVM model, it was noticeable that radial basis function (RBF) kernel was recommended as the kernel function of LS-SVM, since RBF could handle the nonlinear relationships between the spectra and target attributes and give a good performance under general smoothness assumptions. In addition, to achieve the optimal combination of (γ, σ^2^) and avoid overfitting problems, a two-step grid search technique was employed with the leave-one-out cross-validation. The ranges of γ and σ^2^ within 10^−2^–10^5^ were set based on experience and previous researches by our team^25, 26^. The optimal model parameters (γ, σ^2^) were achieved with (33.52, 9.88) based upon the EWs.

**Figure 4:**
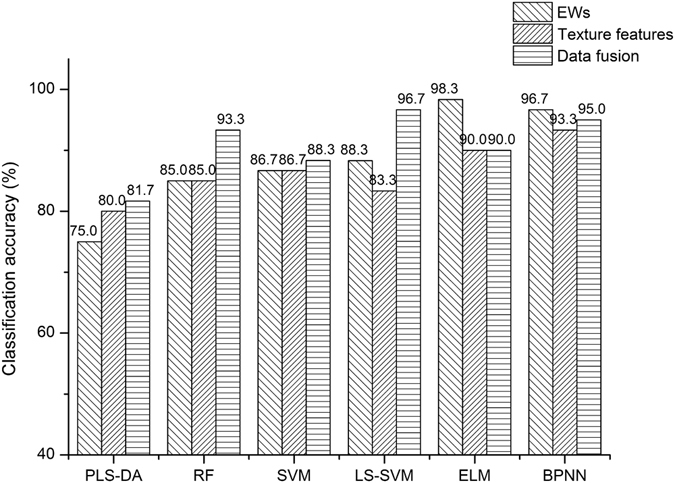
Performance comparison of different machine-learning classifiers including partial least squares-discrimination analysis (PLS-DA), random forest (RF), support vector machine (SVM), back propagation neural network (BPNN), extreme learning machine (ELM) and least squares support vector machine (LS-SVM) with EWs, texture features and data fusion for four-class classification.

The performances of different machine-learning algorithms with combined datasets obtained better results with their classification accuracies of calibration and prediction all above 80% than those only using EWs or texture features (Fig. 4). Although the highest classification rate was not improved but lowered, we believed that this phenomenon was normal because the prediction mechanisms of the classifiers were different. From the perspective of machine learning, when the feature increases, the classification accuracy does not necessarily increase. When combining spectra data and textural features, the BPNN and ELM classifiers might be involved part of noise into the network during their training phase. From our analysis, data fusion overally improved the classification accuracies and received better results by taking advantages of spatial and spectral information of hyperspectral images, which is promising and encouraged for further researches.

Through the observation of other leaves from the infected tobacco plants, the appearance of visual symptoms was found on the 5th day after inoculation, and the typically systemic symptoms of TMV infection (light and dark green areas, and leaf margin deformity) occurred on the 11th day. Therefore, hyperspectral imaging demonstrated a visible difference between the infected (2 DPI) and non-infected (healthy) leaves in a short period of time (i.e. 48 h) in comparison with the reference images (5 days for visible symptoms of infection, 11 days for typical symptoms of TMV infection). Furthermore, the results clearly indicated that hyperspectral imaging with machine-learning algorithms successfully detected the stage of diseased development (2 DPI, 4 DPI or 6 DPI).

## Discussion

We have demonstrated the feasibility and usefulness of hyperspectral imaging in the Vis/NIR spectral region (380–023 nm) for rapid distinguishing TMV-infected tobacco leaves (i.e. 48 h) from healthy samples as well as the degree of disease stage. Coupled with the successive projections algorithm and machine-learning classifiers, correlations were established among the reflectance spectra, texture features and the stage of diseased development. The wavelength number was decreased by more than 98% after wavelengths selection by SPA, which influentially simplified the calibration models and reduced the computation complexity. In addition, it was found that the majority of the EWs selected by SPA for four-class classification were from the spectral reflectance images in the short-near infrared region. The SPA feature selection method together with the ELM classifier resulted in the best overall classification accuracy of 98.33%. The image-based classification results based on the textural features extracted from the images at the optimal wavelengths were comparable to those achieved using the spectral features, with the best overall accuracies of 93.33%. The performance of chemometric models with data fusion manifested better results with classification accuracies of calibration and prediction all above 80% than those only using EWs or texture features; the accuracies were up to 95% employing back BPNN, ELM, and LS-SVM models. Hence, LS-SVM, ELM and BPNN models surpassed other machine-learning classifiers, which provided more alternatives for other researches.

Disease assessment has mostly focused on the detection (diseased and healthy leaves)^15^, identification (i.e. diagnosis of specific symptoms among others, differentiation of various diseases)^13, 14^ and quantification (i.e. measurement of disease severity, e.g. percentage leaf area affected)^10^. However, these studies have either detected diseases when visible symptoms of infection emerged or have not taken into account for developing more robust and accurate machine-learning models. Herein, the hyperspectral line-scan imaging system provided us with excellent detection capabilities, which otherwise cannot be achieved with either imaging or spectroscopy alone. Variable selection algorithm and machine-learning models could be integrated for data mining of HSI. Our results provide compelling evidence for the presymptomatic detection of tobacco disease and classification of three levels of disease degree at early stages, which may mitigate the worldwide losses by TMV and provide a theoretical guidance for crop disease diagnosis and field management.

The classification of hyperspectral images encompasses two main categories: spectral-based classification and image-based classification. Spectral-based classification is to employ spectral features of the mean spectra which are derived by averaging reflectance or transmittance values of the pixels at different wavelengths or the optimal wavelengths provided by variable selection method (e.g. SPA). In the image-based classification, the features from spatial arrangement of pixels and their contextual values, and textural properties are applied as the inputs to the machine-learning classifiers. Extensive literatures are available on the spectral-based classification for disease assessment^27–29^, while image-based classification such as pixel-level processing technique is often neglected. In this study, both spectral features and image textural features at selected wavelengths were used for four-class classification (healthy, 2 DPI, 4 DPI, and 6 DPI). Generally, the spectral data are achieved by the first-order measure of information in the hyperspectral images, while the texture features are extracted by the second-order measures which are according to joint distribution functions. Accordingly, it is reasonable that the spectral information is firstly used to predict attributes and have greater contribution than the texture features in building models. Textural features have achieved excellent results for the qualitative analysis, which are widely used for classification in hyperspectral imaging. The spectra and texture, which reflect different aspects or properties of plants, have complementary advantages and should supplement each other. Consequently, by exploiting spatial and spectral information of hyperspectral images, data fusion is necessary and encouraged in future studies.

A hyperspectral line-scan imaging system was developed to provide excellent detection capabilities, which otherwise cannot be achieved with either imaging or spectroscopy alone. Variable selection algorithm and machine-learning models could be integrated for data mining of HSI. In the light of the present results, it seems feasible to apply HSI as a rapid and accurate alternative to visual assessments, serological and molecular methods. This methodology could be applied on an autonomous agricultural vehicle for reliable and real-time plant disease detection to achieve superior plant disease control and management. Moreover, the realistic application of this trend comes from the fact that different kinds of imaging techniques (e.g. hyperspectral imaging, chlorophyll fluorescence imaging, and infrared thermal imaging) are combined to develop a high-throughput plant phenotyping system to monitor stress levels and physiological states in plants. It is clear that much additional work is required before moving the implementation from near-line application to on-line approach.

## Materials and Methods

### Sample preparation and Infection

Tobacco plants (*Nicotiana tabacum* L. cv. MS Yunyan 87) were grown in an artificial climate chamber of Zhejiang University (120°09′E, 30°14′N) at 24 °C, 50–70% relative humidity and under a 12/12 h light/dark cycle. A total of 180 tobacco plants were used as the experimental materials. Among them 90 tobacco plants were utilized for infection and treatments, and the remaining served as controls. At the 8th leaf stage, plants were transferred to the measuring room for imaging under 12/12 h light/dark. TMV inoculum was obtained by grinding tissue from tobacco plants (susceptible to TMV) infected with TMV strain, which was provided by College of Agriculture & Biotechnology, Zhejiang University; 0.1 g tissue mL^−1^ phosphate-buffered saline (PBS, 0.05 M, pH 7.2). Three or four leaves per plant (*Nicotiana tabacum* L. cv. MS Yunyan 87), were inoculated using the traditionally mechanical method with fine sand as an abrasive. It must be noticed that all plants were sprayed with water mist before inoculation. Finally, inoculated plants and the control group were kept in different growth chambers with the same temperature (24 °C) and humidity (90.0%) and 12 h light/dark cycle.

Before the formal experiment, a preliminary test was carried out and the symptom development of TMV inoculation was daily monitored in the greenhouse. Consequently, the formal experiment was arranged during a week for early detection and classification of tobacco leaves infected with TMV. One leaf collected from per plant was regarded as one sample, and totally 30 samples from healthy leaves and 30 samples from leaves inoculated with TMV were acquired per two days. The collected rape leaves were taken to the laboratory in an incubator filled with ice and cleaned for image acquisition. In total, 180 samples were collected for a period of seven days.

### Hyperspectral image collection and processing

#### Hyperspectral imaging system

**Figure 5:**
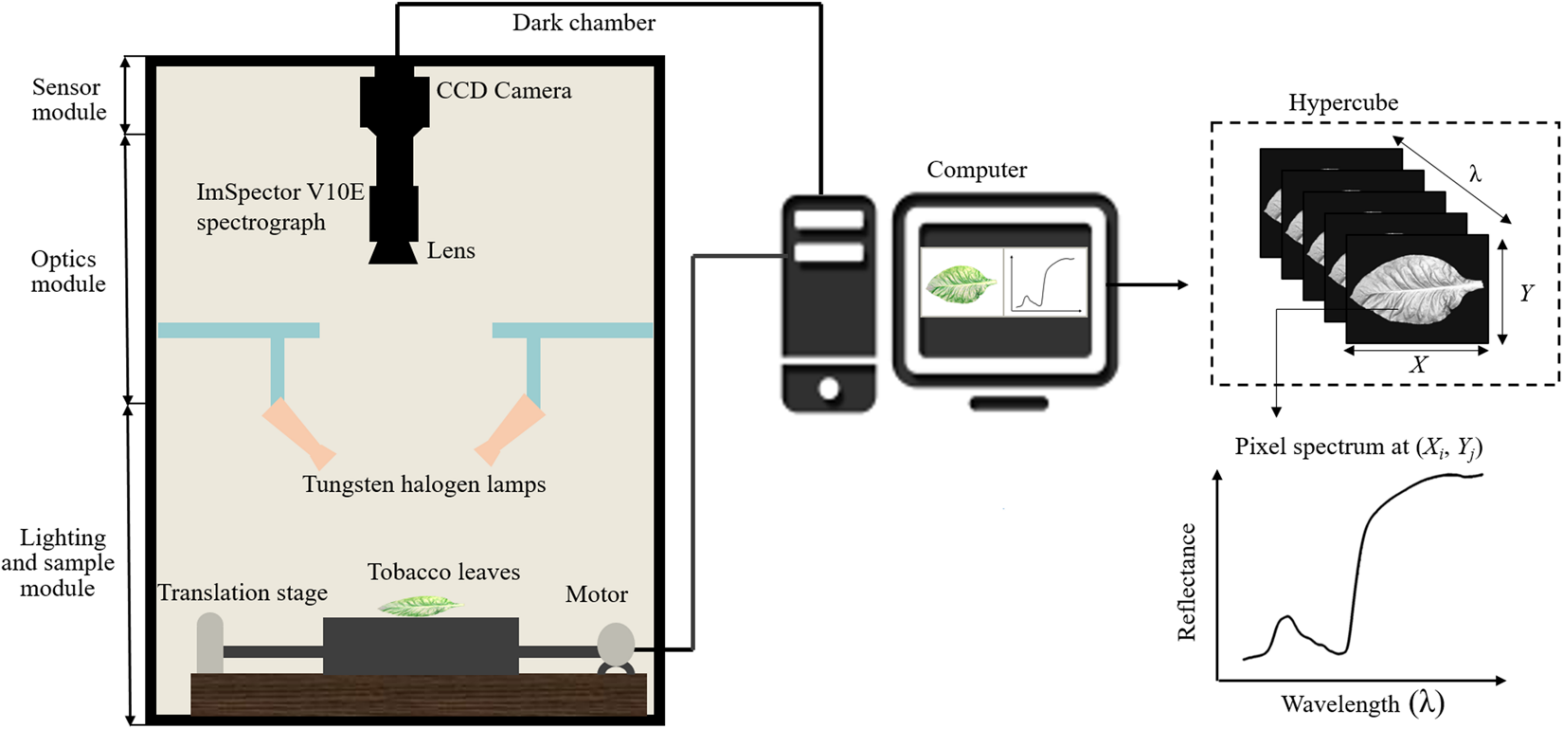
Configuration of the hyperspectral imaging system. The system acquired hyperspectral images at the spectral range of 380–1023 nm with 512 bands.

**Figure 6:**
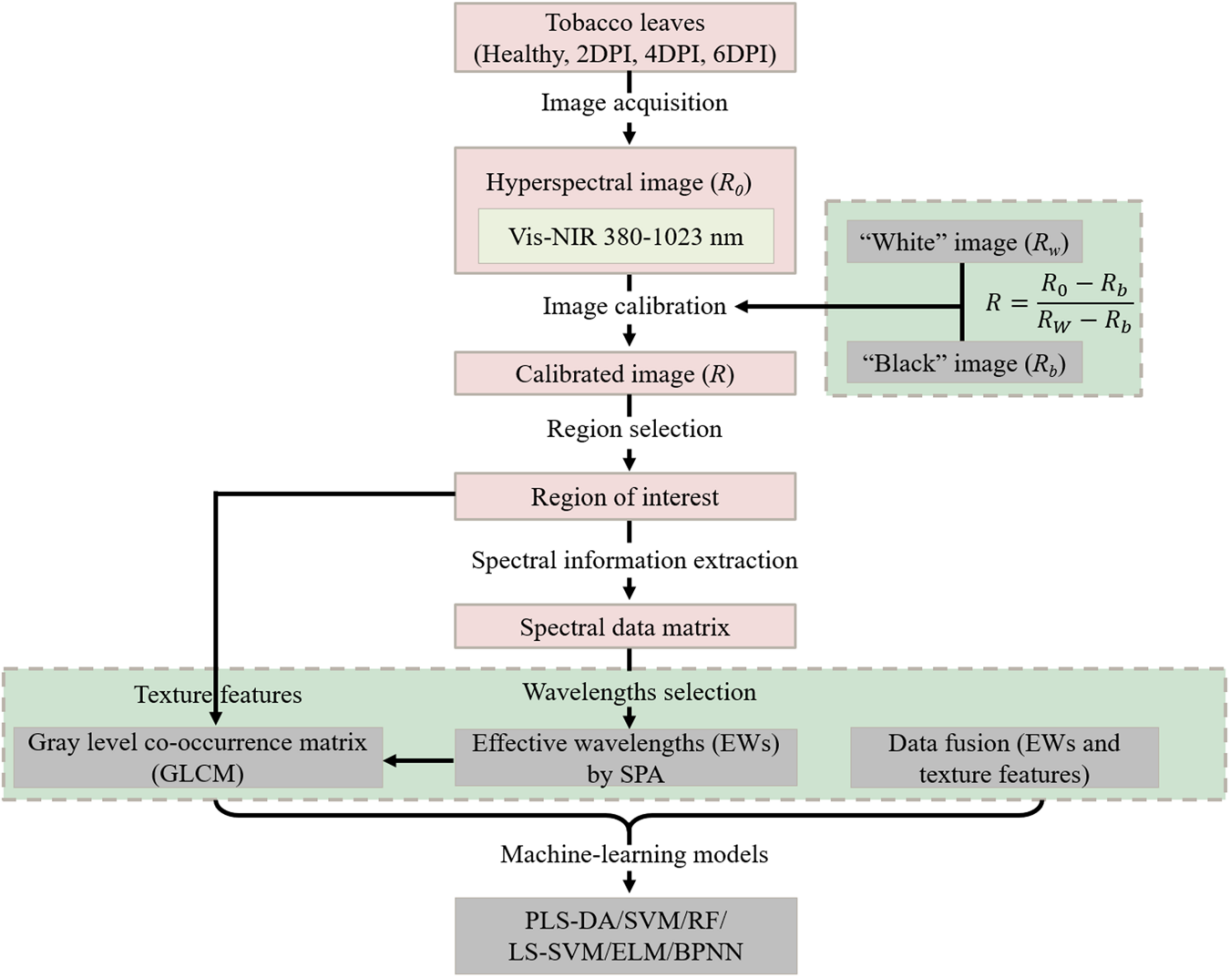
Flowchart of image preprocessing and data analysis for presymptomatic detection of tobacco disease.

Figure 5 illustrates a typical line-scanning configuration (also called ‘Pushbroom’) of hyperspectral imaging system, which records a whole line of an image rather than a single pixel at a time^17^. The system is placed in a dark room, which is composed of the following three modules: (1) Sensor module controls the image acquisition and recording performed with a computer through a data acquisition and system control software (Spectral Image-V10E, Isuzu Optics Corp, Taiwan, China). (2) Optics module is equipped with an imaging spectrograph and a camera. The Vis/NIR spectral range (380–1023 nm with 512 bands) is acquired by an imaging spectrograph (ImSpector V10E, Spectral Imaging Ltd., Oulu, Finland), a 672 × 512 (spatial × spectral) CCD camera (C8484–05, Hamamatsu, Hamamatsu City, Japan) with a camera lens (OLE23, Specim, Spectral Imaging Ltd., Oulu, Finland). (3) Lighting and sample module: an illumination unit which consists of two 150 W tungsten halogen lamps (Fiber-Lite DC950 Illuminator, Dolan Jenner Industries Inc, Boxborough, MA, USA) placed in both sides of the camera symmetrically at an angle of 45° to illuminate the camera’s field of view. The sample is placed on a conveyor belt driven by a stepper motor (Isuzu Optics Corp, Taiwan, China) with an adjustable speed. The flowchart of image preprocessing and data analysis for presymptomatic detection of tobacco disease is presented in Fig. 6.

#### Image acquisition and calibration

The samples were scanned line by line along the *Y*-axis with the sample moving along the *X*-axis at a certain speed to obtain a three-dimensional hypercube, which encompasses both spatial and spectral information where physical and geometric features as well as chemical information could be pulled out. To acquire complete, clear and undistorted images, all image acquisition parameters such as exposure time, height between lens and sample, and motor speed should be adjusted based upon the system configuration. For images at 380–1023 nm, the exposure time, the height and the moving speed were set as 0.09 s, 420 mm, and 3.20 mm/s, respectively. The calibrated image (*R*) is estimated using the following equation:

1 $$R=\frac{{R}_{0}-{R}_{b}}{{R}_{w}-{R}_{b}}$$

Where *R*_*0*_ is the recorded hyperspectral image, *R*_*b*_ is the dark reference image (with 0% reflectance) when the light source is turned off and the camera lens is completely covered with its own non-reflective opaque black cap to remove the thermal activities of the CCD detector, and *R*_*w*_ is the white reference image (Teflon white board with 99% reflectance, 200 mm × 25 mm × 10 mm).

### Spectral information extraction

**Figure 7:**
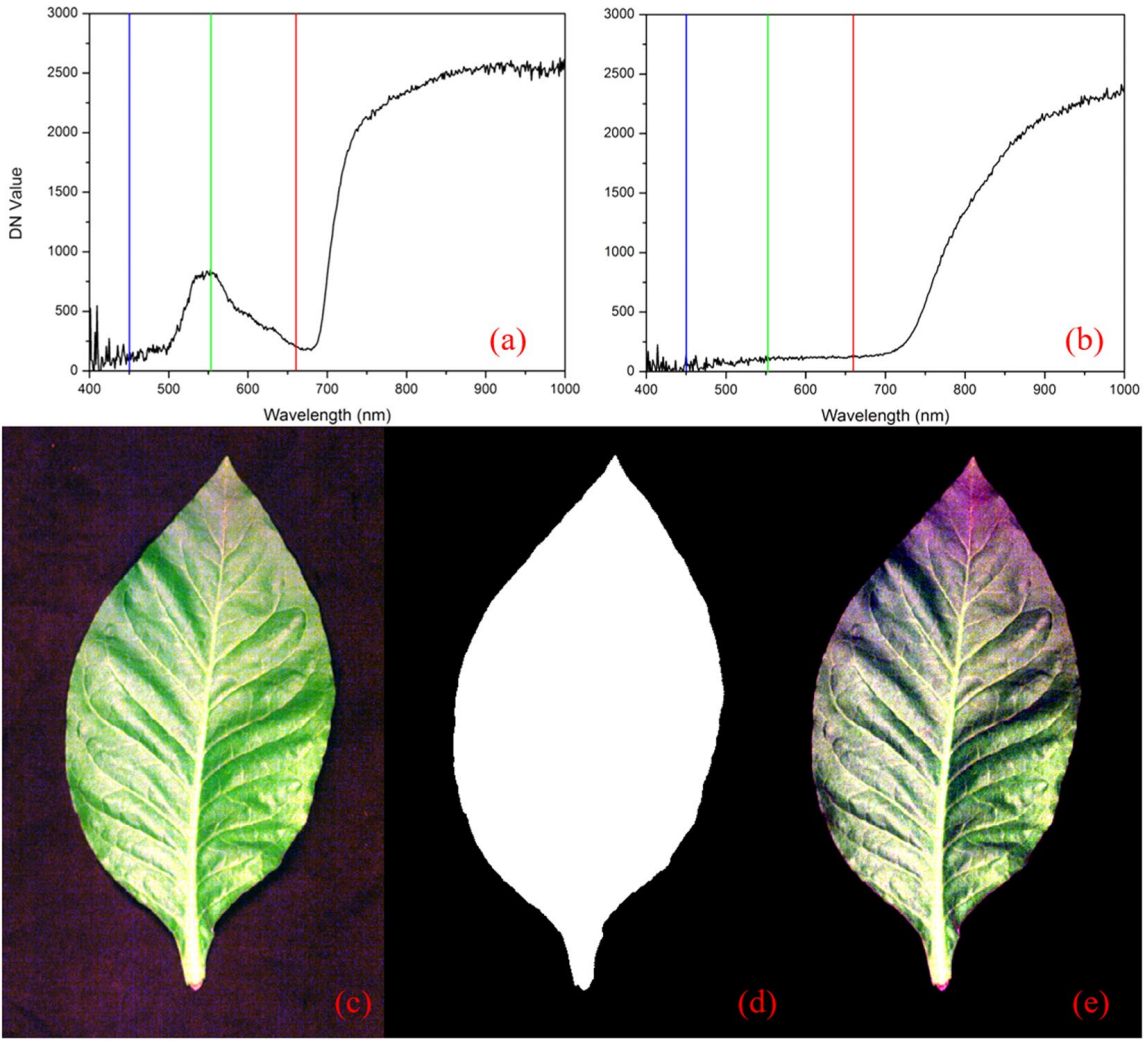
The procedures of image segmentation: (**a**) the spectrum of the sample; (**b**) the background spectrum; (**c**) the calibrated tobacco image; (**d**) build mask to remove background; (**e**) apply mask.

Herein, the segmentation methods were employed based on the image processing technique with the aid of ENVI software, which were developed to isolate the tobacco leaves from the background as detailed in Fig. 7. The key step was to set a proper threshold (Fig. 7a: DN value of sample >300, Fig. 7b: DN value of background <300 at 550 nm) and build mask according to the spectra differences between the sample region and the background at a single waveband (550 nm). The ‘build mask’ is a binary image shown in white pixels (1) and the background is shown in black pixels (0) (Fig. 7d). Then we applied mask to the calibrated image after background removal. The masked image with only tobacco part is indicated in Fig. 7e. The sample region was segmented from background after image segmentation, and region of interest (ROI) was predefined. To reduce the influence of unevenness on plants and deviation of different pixels, a ROI about 900 pixels was selected manually from both sides of each blade and the veins were avoided. For inoculated leaves, the ROIs were located in the symptom areas. Meanwhile the ROIs were in the corresponding areas for healthy samples. Then we used average reflectance spectrum of all pixels within the ROI to represent the sample. Spectra were obtained from all hyperspectral images of leaves and saved in a spectral matrix (*X*). A total of 180 samples were divided into a calibration set and a prediction set with the ratio of 2:1, i.e. one sample was picked out from every three samples consecutively. There were 120 samples for the calibration set (the numbers for healthy, 2 DPI, 4 DPI, and 6 DPI samples are 60, 20, 20, and 20, respectively), and the remaining 60 for the prediction set (the numbers for healthy, 2 DPI, 4 DPI, and 6 DPI samples are 30, 10, 10, and 10, respectively).

### Image textural features extraction

The texture of hyperspectral images could represent the biophysical characteristics (e.g. leaf surface, tissue structure) of plants, including intensities, firmness, color, roughness and their arrangements, which is directly or indirectly related to the growth status of plants. Specifically, the tobacco plants infected with TMV not only lead to the influence on the spectral characteristics, but also result in the change of texture. As one of the most important features in image analysis, texture is characterized by the relationship of the intensities of neighboring pixels^4^, which represents the spatial arrangement of pixel gray levels within the ROI^30^. The most commonly used texture attribute in hyperspectral imaging is the grey-level co-occurrence matrix (GLCM)^30^ which has been applied greatly for quality evaluation and inspection. Herein, four popular textural features, including contrast, correlation, entropy, and homogeneity^21, 31^ were extracted according to GLCM. GLCM is calculated based upon relative distance measured in pixel numbers (*d*) and their relative orientation (*θ*)^19^. Contrast represents the local variations and reflects the image definition in the GLCM. Correlation is the measurement of the linear dependence of the specified pixel pairs. Entropy stands for the image information. Homogeneity measures the closeness of the distribution in an image and indicates the standard of image uniformity. The detailed information and formulas^32, 33^ are defined as follows.

2 $${\rm{Contrast}}=\sum _{i=1}^{G}\sum _{j=1}^{G}{(i-j)}^{2}{P}_{d\theta }(i,j)$$

3 $${\rm{Correlation}}=\frac{{\sum }_{i=1}^{G}{\sum }_{j=1}^{G}(i-Mea{n}_{i})(j-Mea{n}_{j}){P}_{d\theta }(i,j)}{\sqrt{Varianc{e}_{i}}\times \sqrt{Varianc{e}_{j}}}$$

4 $${\rm{Entropy}}=-\sum _{i=1}^{G}\sum _{j=1}^{G}{P}_{d\theta }(i,j)\mathrm{log}\,{P}_{d\theta }(i,j)$$

5 $${\rm{Homogeneity}}=\sum _{i=1}^{G}\sum _{j=1}^{G}\frac{{P}_{d\theta }(i,j)}{1+{(i-j)}^{2}}$$

Where ${P}_{d\theta }(i,j)\,\,$is the number of occurrences of a pair of gray levels *i* and *j*; $Mean=\sum _{i,j=1}^{G}i{P}_{d\theta }(i,j)$, is the average grey level of all pixels; $Variance=\sum _{i=1}^{G}\sum _{j=1}^{G}{(i-u)}^{2}{P}_{d\theta }(i,j)$, is the rate of pixels’ value changes; $u$ is the mean of ${P}_{d\theta }(i,j)$. We extracted the texture features from the gray-scale images at selected EWs suggested by the wavelengths selection method. Four directions (*θ* = 0°, 45°, 90° and 135°) with a uniform distance of *d* = 1 were considered for the extraction and the average values of the four directions were used to represent the textural feature of the sample. The texture features were calculated by a program written in Matlab software.

### Successive projections algorithm and machine-learning classifiers

#### SPA

Hyperspectral images were formed by 512 gray-scale images at 512 wavelength bands in the spectral range of 380–1023 nm. The spectral and spatial information of all 512 bands contained redundancy and collinearity, which was difficult to cope with. Thus, optimal wavelengths selection is very essential for spectral analysis. EWs were selected from the original or preprocessed wavelengths and could be directly used for developing on-line or portable multispectral equipment. Furthermore, EWs could reduce the computation complexity, improve the predictive ability of calibration models, and simplify the calibration models^25^. SPA is a feed forward variable selection method for multivariate calibration, which can greatly reduce the number of variables and improve the speed and efficiency of modeling. Basically, SPA consists of three main steps^34^. Firstly, to select the wavelength variables with minimum collinearity and redundancy as well as maximum projection vector, SPA is conducted by a simple projection in a vector space. Secondly, the EWs are determined according to the minimum RMSEV in the validation set of MLR calibration. The final step aims to remove uninformative variables by a variable elimination procedure without significant loss of prediction capability.

#### Machine-learning classifiers

Since HSI can obtain both spatial and spectral information from an object at the same time, more information was attained compared with other spectral technology. However, because the information from HSI usually had the features of high-dimensions and large data, the suitable machine learning methods for data mining and matrix recognition were necessary. Six supervised classification algorithms including SVM, BPNN, ELM, LS-SVM, PLS-DA and RF were proposed and developed to quantitatively distinguish TMV-infected tobacco leaves from healthy samples as well as the degree of disease stage. SVM finds the hyperplane that gives the largest minimum distance to the training data set, and was implemented according to the quadratic programming optimization with a radial basis kernel^19, 35^. BPNN applies error back-propagation to modify the internal network weights after each training epoch until the goal of the training error or the training epochs of the network is obtained^36^. ELM has good generalization performance for feed forward neural networks with a single hidden layer neural network, which could choose the input weights and the hidden layer biases randomly, and solve problems such as over-fitting and local minima effectively^4, 37^. LS-SVM employs a set of linear equations instead of quadratic programming problems to obtain the support vectors. Moreover, LS-SVM embodies the structural risk minimization principle to avoid overfitting in comparison to traditional empirical risk minimization principle employed by conventional neural networks. There were two significant parameters to be decided in a LS-SVM model. The regularization parameter gam (γ) determined the tradeoff between minimizing model complexity and minimizing the training error. The parameter sig^2^ (σ^2^) of RBF kernel function was the bandwidth, which implicitly defined the nonlinear mapping from input space to some high dimensional feature space^25^. PLS-DA is a widely supervise classifier based on PLSR, with integers representing the categories as Y. The outputs of PLS-DA are real numbers with decimals^38^. RF is an ensemble algorithm with a multitude of decision trees. The samples trained in each decision tree are randomly selected from the training set by recovery sampling for developing a random forest^39^.

Because of the EWs selection, there was no significant difference on the computation time of various machine-learning models for classifying healthy and TMV-infected (2 DPI, 4 DPI, and 6 DPI) leaves. Hence, the capabilities of the six classifiers were assessed mainly by the accuracy of each class and the overall accuracy. The classifier parameters were estimated for multiple datasets, possessing variation in data analysis (EWs, texture features and data fusion). Spectral data extraction was conducted on ENVI 4.6 (ITT, Visual Information Solutions, Boulder, USA). All computations and machine-learning algorithms were performed with the aid of chemometric software Unscrambler® 10.1 (CAMO AS, Oslo, Norway) and Matlab R 2009b (The Math Works, Natick, USA).
